# Editorial: Women and clinical microbiology 2021

**DOI:** 10.3389/fcimb.2022.999967

**Published:** 2022-10-21

**Authors:** Krisztina M. Papp-Wallace, Shannon D. Manning, Arryn Craney, Masae Kuboniwa, Sophia Vourli

**Affiliations:** ^1^ Research Service, Veteran Affairs Northeast Ohio Healthcare System and Department of Medicine, Case Western Reserve University, Cleveland, OH, United States; ^2^ Department of Microbiology and Molecular Genetics, Michigan State University, East Lansing, MI, United States; ^3^ Orlando Health, Orlando, FL, United States; ^4^ Department of Preventive Dentistry, Osaka University Graduate School of Dentistry, Suita, Osaka, Japan; ^5^ Laboratory of Clinical Microbiology, Attikon University Hospital, School of Medicine, Department of Microbiology, National and Kapodistrian University of Athens, Athens, Greece

**Keywords:** female, vaginitis, pregnancy, surveillance, reproduction, Candidiasis, microbiome, SARS- CoV-2

*Women and Clinical Microbiology 2021* is an inaugural Research Topic in Frontiers in Cellular and Infection Microbiology Clinical Microbiology aimed at commemorating the accomplishments of women in science by compiling articles focused on women’s health or research studies conducted by women as a primary or corresponding author. This Research Topic was chosen because less than 30% of researchers worldwide are women, which is likely due to long-standing biases and gender stereotypes that have discouraged women from entering science-related fields. To help promote gender equality and overcome these stereotypes, Frontiers in Cellular and Infection Microbiology introduced this topic to promote research by women scientists studying cellular and infection microbiology. The nine publications, 8 research studies and 1 minireview, presented by women scientists highlight the diversity of research performed across the entire breadth of clinical microbiology, from female reproductive health to diagnostic approaches and beyond.

Four manuscripts (Gao et al., Li et al., Huang et al., and Lu et al.) tackled different issues in relation to maternal health during pregnancy. One study by Gao et al. compared the Copan Walk Away Specimen Processor (WASP), an automated diagnostic tool, to conventional manual culture methods. The research team used this tool to detect pathogens such as *Chlamydia* spp., *Neisseria gonorrhoeae*, *Candida albicans*, group B *Streptococcus* (GBS), and *Ureaplasmaurealyticum*, from swabs collected from the lower reproductive tract of pregnant women. Because the presence of these pathogens can lead to perinatal complications, including death of the fetus, accurate identification of these microbes is critical. The data revealed that conventional methods could distinguish more pathogen types compared to WASP; however, WASP was better at detecting single colonies. The Copan swabs were also tested using fluorescence-based qPCR and immunochromatography to identify *Chlamydia trachomatis*, but only the qPCR approach worked. Since the Copan WASP has been successfully applied to other specimen types (e.g., respiratory tract), there is potential for this approach in women’s health. Indeed, the quick turnaround time as well as the use of less resources (e.g., reagents, personnel) makes automated diagnostics a worthy pursuit.

A second maternal health study, which was conducted by Huang et al., addressed the relationship between the microbiota and pregnancy complications. To explore the mechanisms linked to increased risk of complications in women with advanced maternal age (AMA), the vaginal and intestinal microbiota were characterized in three groups of women. These groups included pregnant women with AMA over 35 years of age, pregnant women of non-advanced maternal age (NMA) less than 35 years, and non-pregnant women over the age of 35 (control group). The results showed that the AMA group had significantly increased α-diversity and decreased β-diversity as well as enriched *Bifidobacterium* and decreased *Lactobacillus johnsonii* in the vaginal microflora compared to NMA group. As *L. johnsonii* can inhibit the growth of pathogens and reduce the incidence of preterm birth, the authors suggested that decreased levels were associated with an increased risk of complications during pregnancy in women with AMA. A third study by Li et al. evaluated how clinical and microbiological characteristics of mixed vaginitis in the third trimester impacted adverse pregnancy outcomes, which is an understudied area of research. Over 1,600 women in late-stage pregnancy were included in this analysis and 66 had mixed vaginitis. Independent risk factors for developing mixed vaginitis included previous vaginitis during pregnancy, positive glucose tolerance test during pregnancy, sex during pregnancy, and history of genital infection before pregnancy. The main adverse outcome for pregnant women with mixed vaginitis, however, was found to be peripartum infection.

In the fourth manuscript relevant to maternal health, Lu et al. examined the role of human milk lactoferrin in the prevention of GBS disease by investigating its antimicrobial and anti-biofilm activity against a diverse set of 25 phenotypically and genetically diverse isolates. Importantly, lactoferrin showed growth inhibition and anti-biofilm activity against 14 and 21 of the clinical GBS isolates, respectively. The maternal colonizing GBS isolates were more susceptible to inhibition than the neonatal invasive isolates. In addition, variation in susceptibility was observed across multilocus sequence types when treated with 750 µg/mL of lactoferrin, while different capsular types had differences in susceptibility at 250 µg/mL of lactoferrin. Lactoferrin also had better anti-biofilm activity against isolates with enhanced biofilm production, highlighting its potential for reducing bacterial densities and subsequently limiting transmission to neonates.

Other areas of focus within this topic include molecular surveillance (Boucherabine et al.), the development and validation of a rapid predictive risk scoring function (Zhang et al.), and the analysis of a diagnostic approach (Senok et al.). To begin with, Boucherabine et al. reported the potential of health care worker (HCW) mobile phones and environmental surfaces to serve as reservoirs for SARS-CoV-2 and multidrug-resistant superbugs. The point prevalence study was conducted in the emergency room, which was divided into a non-COVID-19 patient zone and a zone dedicated to confirmed or suspected COVID-19 patients. Three methicillin-resistant *Staphylococcus aureus* isolates and one pan-drug resistant carbapenemase-producing *Acinetobacter baumannii* isolate were recovered from mobile phones. A shotgun metagenomics analysis revealed that a total of 76 and 46 different antibiotic resistance genes were found in the mobile phone and environmental samples, respectively. In addition, one mobile phone from the COVID-19 zone tested positive for SARS-CoV-2, demonstrating that phones serve as fomites and may contribute to the transmission of clinically relevant bacterial and viral pathogens. The authors therefore recommended the introduction of highly efficient phone sanitization methods in hospitals and public areas.

The aim of Zhang et al.’s prospective cohort study was to develop and validate a risk score to predict invasive candidiasis (IC) in intensive care unit (ICU) patients by evaluating clinical risk factors as well as lymphocyte subtyping. IC is highly prevalent in ICU patients and outcomes are poor especially if the initiation of antifungal therapy is delayed. A total of 1054 ICU patients were included of which 69 had IC and were included in the analysis. Multivariate logistic regression was used to identify which clinical risk factors and lymphocyte subtypes significantly correlated with IC. Notably, a CD8+ T-cell count ≤143 cells/mm^3^, receipt of high-dose corticosteroids (dose ≥50 mg prednisolone equivalent), receipt of carbapenem/tigecycline, APACHE II score ≥15, (1,3)-β-D-glucan (BDG) positivity, and emergency gastrointestinal/hepatobiliary (GIT/HPB) surgery were highly predictive for rapid diagnosis of IC in ICU patients. Risk scores were also stratified into three different cohorts based on the former factors. Together, this risk assessment has the potential to be useful to predict IC in ICU patients.

In another study, Senok et al. evaluated a novel lateral flow assay for the detection of Panton Valentine leukocidin (PVL), a virulence factor that can impact the severity of Staphylococcal infections. *S. aureus* isolates from 129 patients were screened for PVL by the lateral flow assay and molecular detection of *pvl*. Excellent correlation was observed between the new PVL assay and molecular testing as 76 isolates were negative by both molecular and antigen testing, while 53 isolates were positive by both molecular and antigen testing. However, the authors noted 4 isolates with weak antigen positivity and as a precaution state a diagnostic sensitivity of 92.5%, a negative predictive value of 95% as well as specificity and positive predictive value of 100% can be expected. This work supports the implementation of an accurate assay for PVL detection from Staphylococcal isolates diagnostically. It will be interesting to see in the future the clinical utility of this assay.

A description of initial studies that can aid in the development of a vaccine (Urrea-Quezada et al.) and a minireview (Moubareck and Halat) were also included in this Research Topic. The work of Urrea-Quezada et al. focused efforts on the gp15 protein as a novel vaccine target for Cryptosporidium, a parasitic cause of gastroenteritis that can be severe in children and the immunocompromised. To identify immunogenic candidate peptides, a library of 5 synthetic peptides (4 against gp15 and 1 against gp40) was created and screened for IgM and IgG responses in serum collected from 39 human cases of cryptosporidiosis and 90 healthy individuals as controls. In comparison to the control group, the authors observed a statistically significant response in two of the synthetic peptides (A133 and A32) designed from gp15. This strong antibody response supports the further investigation of gp15 as a vaccine candidate for cryptosporidiosis. Finally, the mini-review by Moubareck and Halat summarized studies dealing with the link between COVID-19 infections and carbapenemase-producing *Enterobacteriaceae* and *Acinetobacter*. Concerning *Enterobacteriaceae*, their search revealed reports showing increases in colonization or infection with carbapenemase-producing *Enterobacteriaceae* in COVID-19 versus non-COVID-19 patients. Furthermore, they highlighted reports showing a marked increase in carbapenemase-producing *Enterobacteriaceae* in regions with recent decreases of such organisms, or a shift in carbapenemase-producing *Klebsiella pneumoniae* dominant genetic types. Similarly, they highlighted studies reporting an increase in infections caused by carbapenem-resistant *A. baumannii* in COVID-19 patients, identifying *A. baumannii* as the most common pathogen isolated from COVID-19 patients, with carbapenem resistance rates above 90%. Together, these studies further demonstrate the need for antibiotic stewardship and widespread surveillance for carbapenemase-producing pathogens in order to contain their spread.

In conclusion, this inaugural Research Topic highlighted the impactful research being conducted by women scientists around the world. Not only are they advancing the field of clinical microbiology in terms of women’s health, but in other important areas as well.

## Author contributions

KP-W, SM, and SV participated in the editorial review process for these manuscripts. KP-W, AC, MK, and SV wrote the first draft the manuscript. All authors contributed to manuscript revisions, read, and approved the submitted version.

## Funding

This work was supported in part by funds and/or facilities provided by the Cleveland Department of Veterans Affairs to KMPW, the Veterans Affairs Merit Review Program Award 1I01BX002872 to KMPW from the Biomedical Laboratory Research & Development Service of the VA Office of Research and Development. SM is funded by the MSU Foundation. MK is supported by JSPS KAKENHI Grant Numbers JP22H03300 and JP22K19622.

## Conflict of interest

The authors declare that the research was conducted in the absence of any commercial or financial relationships that could be construed as a potential conflict of interest.

## Publisher’s note

All claims expressed in this article are solely those of the authors and do not necessarily represent those of their affiliated organizations, or those of the publisher, the editors and the reviewers. Any product that may be evaluated in this article, or claim that may be made by its manufacturer, is not guaranteed or endorsed by the publisher.

## Author disclaimer

The contents do not represent the views of the U.S. Department of Veterans Affairs or the U.S. Government.

